# Self-structuring foods based on acid-sensitive low and high acyl mixed gellan systems to impact on satiety

**DOI:** 10.1016/j.foodhyd.2013.07.014

**Published:** 2014-03

**Authors:** Jennifer F. Bradbeer, Robin Hancocks, Fotios Spyropoulos, Ian T. Norton

**Affiliations:** Department of Chemical Engineering, University of Birmingham, Edgbaston, Birmingham B15 2TT, UK

**Keywords:** Acid gelation, Low acyl gellan gum, High acyl gellan gum, Mixed gel structure

## Abstract

This study investigated the *in vitro* acid-induced gelation of mixed systems of two biopolymers; low acyl and high acyl gellan gum. Rheological and texture analysis showed that these mixed gels displayed textures that lay between the material properties exhibited for the low and high acyl variants. DSC analysis showed that mixtures of the low acyl and high acyl forms exhibit two separate conformational transitions at temperatures coincident with each of the individual biopolymers.

Various metabolically relevant pH environments and hydrocolloid concentrations were investigated. These resulted in very different acid gelled structures, which were characterised by texture analysis. The structures of the acid gels were shown to depend upon the pH, hydrocolloid concentration and proportion of each biopolymer used during their production.

A selection of these mixed gellan structures were assessed post-production in terms of their response to prolonged exposure to an acidic (pH 1), stomach-like, environment. This resulted in a significant increase in the gel strength, regardless of the biopolymer proportions. The high acyl gellan was less acid-sensitive, and subsequently no evidence of acid gelation was observed with high acyl gellan at a proportion greater than 60% of the total biopolymer.

The findings presented here demonstrate that structuring as well as de-structuring of mixed gellan acid gels can be controlled in acidic environments similar to those that are present in the stomach after food consumption.

## Highlights

• Mixed systems of low acyl and high acyl gellan gum were prepared.
• The mixed gels had properties intermediate to either variant alone.
• *In vitro* acid-induced gelation of the mixed systems was investigated.
• Structures were dependent on pH, hydrocolloid concentration and biopolymer fraction.
• Post-production exposure to an acidic environment increased gel strength markedly.

## Graphical abstract

**Figure:**
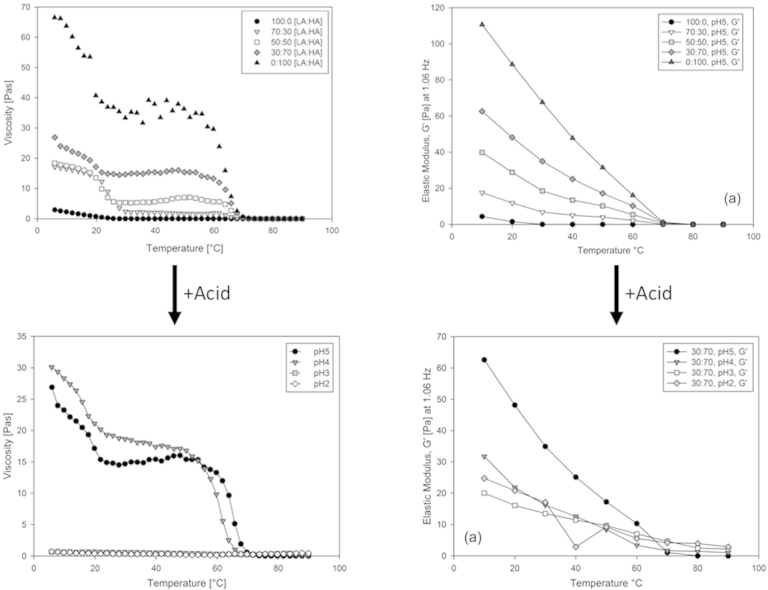

## 1 Introduction

Before the 20th century, obesity was rare (Haslam, 2007). In 1997 however, the World Health Organization (*WHO*) formally recognized obesity as a global epidemic (Caballero, 2007). As of 2008 the *WHO* estimated that globally at least 500 million adults are obese, with higher rates among women than men. Once considered a problem only of high-income countries, obesity rates are rising worldwide and affecting both the developed and developing world (Tsigosa et al., 2008).

One way of tackling the high and rising levels of morbid obesity in today's society is to control the consumer's energy intake from foods. One problem is that foods have become softer, more easily digestible and therefore less satiating. This leads to the individual feeling hungry more quickly and subsequently wanting to eat again, often between meals. Therefore, there is a requirement to find ways to control the rate of food digestion without impacting on the enjoyment of the food. This needs to be both in terms of its digestive properties and the sensory aspects it delivers during consumption. In addition, this needs to be achieved in such a way that the food is stable during storage and distribution (Norton, Fryer, & Moore, 2006; Norton, Moore, & Fryer, 2007).

One approach, that could impact on people's appetite and can be delivered in liquid or soft solid products without an adverse effect on consumer response, is the use of hydrocolloids that respond to the pH conditions experienced inside the stomach. Once in contact with low pH conditions these hydrocolloids self-structure to form gels. It is well known that alginate gels as its pH is lowered (Draget, Skjåk-Bræk, & Stokke, 2006), and previous studies have investigated the ability of alginate to structure the stomach contents (Hoad et al., 2004; Norton, Frith, & Ablett, 2006). These studies showed that some structuring occurred within the stomach, as measured by NMR imaging, and that satiation was prolonged as a result. Their findings highlight the need for further study, as it was reported that acid gels were produced too quickly after entering the stomach and thus gave less than optimal structuring of the stomach contents. In addition, the alginate used was sensitive to calcium ions and so would prove to be very difficult to incorporate into foods.

One alternative hydrocolloid that forms acid gels is gellan gum (Yamamoto & Cunha, 2007). This has received little attention and has only been widely investigated under slow acid release from Glucono-Delta-Lactone (GDL), with just a few exceptions that have studied acid gelation by direct addition of acid, which is more relevant to digestion (Norton, Cox, & Spyropoulos, 2011). Gellan gum is a carboxylated extracellular polysaccharide secreted by the organism *Sphingomonas elodea*during aerobic fermentation (Kang & Veeder, 1982). The gellan polymer consists of monosaccharides β-D-glucose, β-D-glucuronic acid and α-L-rhamnose in molar ratios of 2:1:1 (Sanderson, 1990) linked together to form a primary linear structure. The biopolymer is produced with two acyl substituents present on the 3-linked glucose, L-glycerol positioned at O(2) and acetyl at O(6). Direct recovery of the polysaccharide from the fermentation broth yields the high acyl form whereas deacylation by alkali treatment results in the low acyl variant. Gellan gum is currently commercially available in both the high acyl and the low acyl form. When hot solutions of gellan gum are cooled in the presence of gel-promoting cations, gels of various textures can be formed, principally through cation-mediated helix–helix aggregation (Gibson & Sanderson, 1997). Intermediate mechanical properties between those of the individual components are seen when combining low acyl gellan with high acyl gellan to form mixed gels (Sanderson, 1988).

The aim of this work was to investigate the acid gelation of gellan in order to gain an insight into the gel structures that can be produced by direct addition of acid, as well as investigating the effect of mixing the two variants of gellan to control the mechanical properties of the acid gel. Both the effects of total polymer concentration and the ratio of low to high acyl gellan on the mechanical properties of the mixed gellan gels were investigated. The structure of the produced acidified mixed gels was studied by means of texture analysis, rheology and differential scanning calorimetry. Mixed gellan gels were assessed post-production in terms of their response to a prolonged exposure to an acidic environment that simulates the conditions found in the stomach after ingestion.

## 2 Experimental

### 2.1 Materials

Low acyl gellan gum and high acyl gellan gum (Kelcogel F and Kelcogel LT100), provided by CPKelco (UK). The water used was filtered through a reverse osmosis milli-Q water system. HCl acid (38% wt./wt.) was purchased from Fisher Scientific (Loughborough, UK), and diluted to form a 0.5% (0.137 mol/dm^3^) stock solution, which was used for the direct acidification of all the produced acid gel structures. All materials were used with no further purification or modification of their properties.

### 2.2 Methods

#### 2.2.1 Preparation of mixed gels

Aqueous mixed hydrocolloid solutions of the two gellan variants with total concentrations between 0.5 and 3 wt.% were prepared by dissolving the required amounts of each hydrocolloid in distilled water at 80 °C. The natural pH of the gellan solutions was measured as 5.4, which was not dependent on concentration. The pH of the mixed gellan solutions was adjusted by drop wise addition of 0.5 wt.% (0.137 mol/dm^3^) HCl (also at 80 °C) and these acidified solutions were then poured into cylindrical moulds (22.5 mm internal diameter and 50 mm height), which were stored at room temperature for 24 h to allow for gel formation. Texture analysis of all mixed acid gel samples was carried out immediately after the 24 h setting period.

#### 2.2.2 Texture analysis

The structure of the prepared mixed acid gels was assessed by performing compression tests using a TA XT plus Texture Analyser (Stable Micro Systems Ltd., UK), with a 40-mm diameter cylindrical aluminium probe. All samples had a diameter of 22.5 mm and their length when measured was 10 mm. All measurements were carried out in triplicate with a compression rate of 1 mm/s.

The force/distance data, as obtained directly from the texture analyser, was converted into true strain (*ɛ_H_*) and true stress (*σ_T_*) data using the following equations (Moresi & Bruno, 2007):

(1) $${ɛ}_{E}=\frac{H_{0}-h}{H_{0}}$$

(2) $${ɛ}_{\text{H}}=\text{ln}\left(1+{ɛ}_{E}\right)$$

(3) $$\sigma_{E}=\frac{F}{A_{0}}$$

(4) $$\sigma_{T}=\sigma_{E}\left(1+{ɛ}_{E}\right)$$

where *ɛ*_*E*_ and *ɛ*_*H*_ are the engineering and true (Hencky) strain, *σ*_*H*_ and *σ*_*T*_ are the engineering and true stress, *H*_0_ and *A*_0_are the initial height and cross-sectional area of each sample and *F* and *h* are the compression force applied and height of each sample as recorded during the compression tests.

From the obtained true stress/true strain curves, the slope of the initial linear region (up to strain values of 0.05%) can be used to calculate the Young's modulus (Smidsrød, Haug, & Lian, 1972) while the slope of the second linear region (for strain values over ∼0.1%), leading to the subsequent structure failure can be used to calculate the bulk modulus (Nussinovitch, 2004). These calculated moduli provide information regarding the two deformation mechanisms associated with each of the two linear regions. When the samples are initially loaded the connections between the hydrocolloid molecules within the gel network are deformed, as a result of the applied stress. During this initial compression stage the gel matrix exhibits elastic behaviour, the measure of which is given by the calculated Young's modulus. When a critical stress is reached the connections in the hydrocolloid network break where the process of deformation enters a second much steeper linear region. During this compression stage the exhibited behaviour is non-elastic and the slope of the linear region in the true stress/true strain curve, thus the calculated bulk modulus, relates to the stiffness/deformability of the gel matrix, until structure failure occurs.

**Fig. 1:**
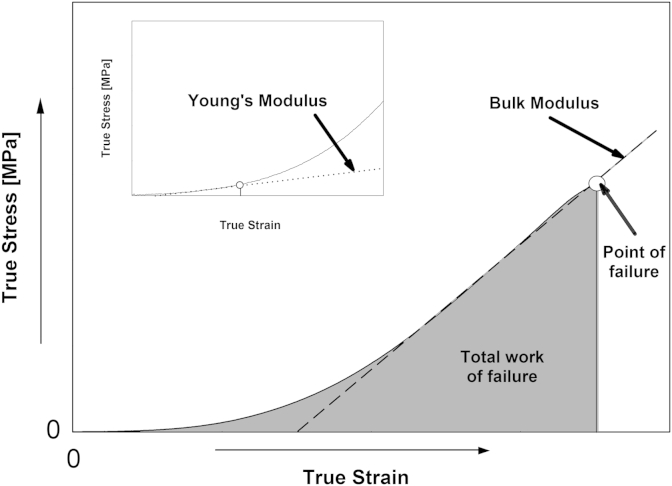
A typical true stress/true strain curve obtained during uniaxial compression of gellan gum acid gels adapted from Fig. 1, Norton et al., 2011. Also shown is how the data in the plot is interpreted to give the Young's modulus, bulk modulus and total work to failure for the acid gel structures.

Finally the total work to failure (Kaletunc, Normand, Nussinovitch, & Peleg, 1991) is the total work (given as work per unit area in this study) that is required in order for the structure to fail and this is determined from the area, up to the point of failure, under the true stress/true strain curve. Fig. 1 shows a typical true stress/true strain curve and also how the data in the plot is interpreted to give the Young's modulus, bulk modulus and total work to failure for the acid gel structures.

#### 2.2.3 Post-production exposure to an acidic environment

The 3 wt.% mixed gellan gels were exposed to an acidic environment by placing the 10 mm samples into an acid solution (pH 1) for periods of time of up to 3 h 3 h was an exposure time chosen as an average gastric residence time although residence times as short as 1 and as long as 5 h have been reported depending on circumstances (Mojaverian et al., 1985). Texture analysis of these samples was performed immediately after exposure. Experiments were performed in triplicate to ensure reproducibility.

#### 2.2.4 Rheological analysis

Rheological measurements on the aqueous mixed gellan solutions in their natural and pH adjusted forms were carried out using a Gemini HR nano stress-controlled rheometer using a 4° truncated cone and plate geometry, 40 mm in diameter. A cone and plate geometry was selected as it provides a well-defined and uniform shear field during experimentation. The aqueous mixed gellan samples were heated to 90 °C, before loading on to the pre-heated plate of the rheometer. All experiments were carried out using a silicon oil moisture barrier to reduce evaporation.

A constant shear rate of 0.5 s^−1^ was applied whilst cooling at 2 °C/min^−1^–5 °C to obtain viscosity. Frequency response in the range 0.1–10 Hz was obtained under constant stress (0.1 Pa) at 10 °C intervals from 90 °C to 10 °C.

#### 2.2.5 Differential scanning calorimetry

Differential scanning calorimetry measurements were performed using a Setaram μ-DSC. Stainless steel sample vessels were filled with 0.76 g of the sample solution and the water-filled reference vessel matched to this within 1 mg. At the beginning of each experiment both sample and reference were heated to 95 °C to eliminate errors caused by thermal history during sample preparation and loading. A constant rate of 0.2 °C/min^−1^ heating and cooling was used in these experiments.

## 3 Results and discussion

### 3.1 Characterisation of mixed gellan gels

Mixed gellan gels were produced to 3 wt.% total biopolymer, by combining the low and high acyl gellan variants in different ratios.

**Fig. 2:**
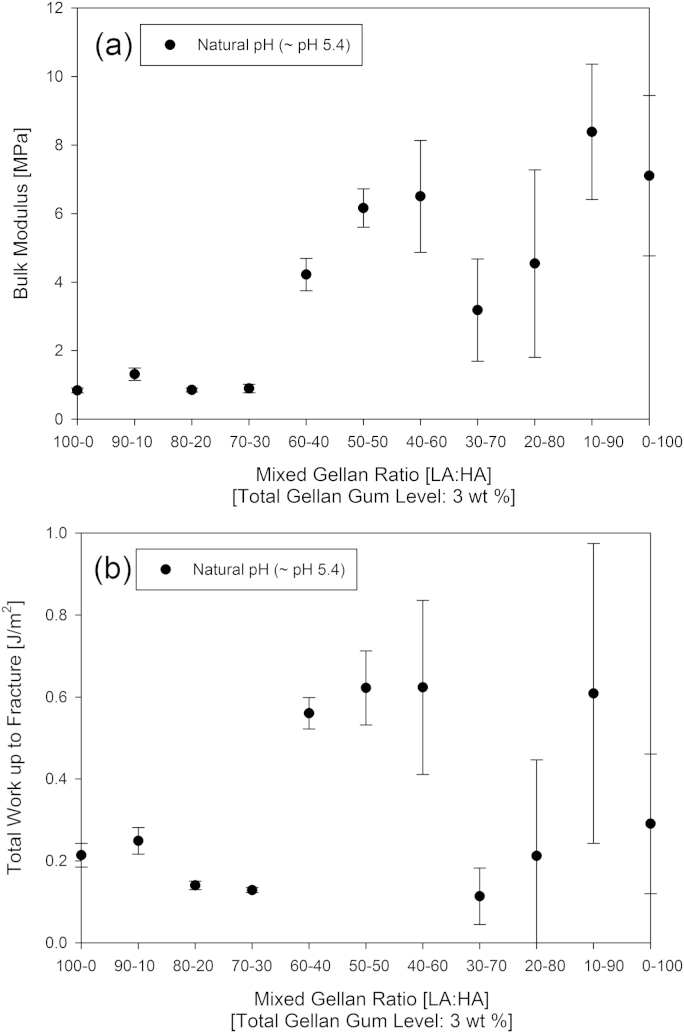
Bulk modulus (a) and work up to fracture (b) for 3 wt.% mixed gellan gels as a function of increasing high acyl gellan percentage (0–100% of the total biopolymer added).

Texture analysis (Fig. 2) shows that the ratio of HA:LA variants affected the resulting gel structure. The mechanical properties of the mixtures depend on the ratio of the two biopolymers used. At natural pH mixtures with high acyl gellan weight fractions >30% produce higher bulk moduli gels than those with lower high acyl content. The gels in Fig. 2 display mechanical properties between those exhibited for the high and low acyl gellan variants, despite the high acyl gellan used behaving differently to that reported in literature (Sanderson, Bell, Clark, & Ortega, 1988). These results show a constant bulk modulus and total work up to fracture with high acyl proportions from 0% to 30%, indicating that the addition of high acyl gellan in this range is not changing the overall structure; this implies that up to this point the gel structure is phase separated with the low acyl form continuous.

Between 30% and 60% high acyl gellan proportion, the bulk modulus and work to fracture results increase gradually, reaching a maximum at around 60% high acyl gellan variant. This suggests that the biopolymers are now forming an interpenetrating network structure. Past this maximum the bulk modulus and work to fracture results fall dramatically with very low values at 70 and 80% high acyl gellan. This suggests a phase separation at these values, with neither variant able to form a continuous matrix, leading to a weak gel structure.

At 90% high acyl gellan proportion and above, the high acyl gellan is continuous, with rheology the same as (bulk modulus and work to fracture within error bounds) the rheology of the high acyl variant alone.

This is in contrast to the smooth increase of bulk modulus values observed by Morrison, Sworn, Clark, Chen, & Talashek (1999) as the proportion of high acyl gellan was increased.

**Fig. 3:**
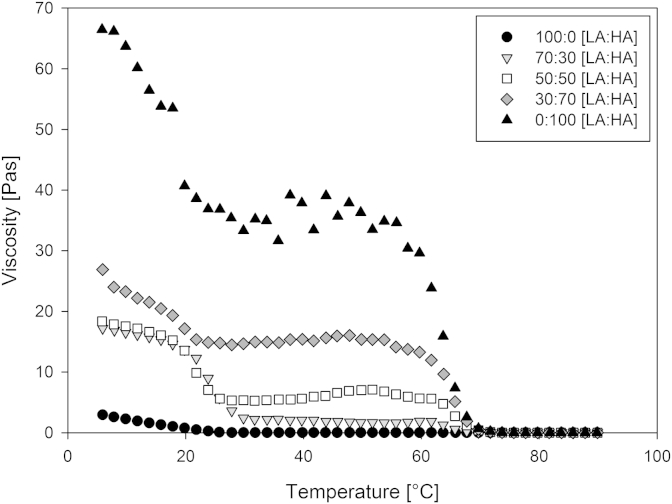
Viscosity measurements of 0.5 wt.%, pH5 mixed gellan aqueous solutions during a temperature ramp (90–5 °C) at 2 °C/min, 0.5 s^−1^ constant shear rate.

To further investigate the properties of the mixed gels, viscosity measurements were taken for 5 of the mixed gellan weight ratios. A temperature ramp from 90 to 5 °C was performed using a cooling rate of 2 °C/min^−1^ whilst keeping a constant shear rate of 0.5 s^−1^. Fig. 3 shows the viscosity of 0.5 wt.% mixed gellan aqueous solutions at their natural pH.

Increasing the proportion of high acyl gellan results in a subsequent increase in viscosity. The rheological onset of gel formation on cooling is apparent for both of the gellan types within the mixed systems at their respective transition temperatures of approximately 25 °C (LA) and 65 °C (HA). This agrees with the rheological measurements taken from mixed gellan systems by Kasapis et al., (1999), who on cooling from high temperature observed two regions of steep increase in G′, the first coincident with the sol–gel transition of high acyl gellan at high temperature and the second with the corresponding transition of the deacylated polymer at much lower temperature.

The mixed gellan solutions with 30–70% high acyl gellan each show two distinct coil-helix transition temperatures on cooling for the high acyl and low acyl gellan variants, which shows that the problems associated with the high gelation temperature of the high acyl gellan gum are present in the mixed systems.

At 100% high acyl gellan the coil-helix transition of the high acyl gellan is at approximately 65 °C, but a second steep transition is also observed at around 25 °C. The first step at 65 °C corresponds to the conversion of the polymer from the disordered coil state to the double-helix form (Chandrasekaran & Radha, 1995; García, Alfaro, Calero, & Muñoz, 2011; Yamamoto & Cunha, 2007). However, conformational ordering does not, in itself give a cohesive network. Therefore, a possible explanation for the appearance of the low temperature transition at 100% high acyl gellan is that a delayed salt ordering (Miyoshi, Takaya, & Nishinari, 1995; Ogawa, Matsuzawa, & Iwahashi, 2002) is occurring following the initial high acyl gellan conformational ordering. This phenomenon occurs most strongly in high acyl gellan systems, whereby the high coil-helix transition temperature is distinct from the salt ordering at lower temperatures, unlike low acyl gellan. Additionally, the L-glycerol groups in high acyl gellan increase the stability of the double helix by forming additional hydrogen bonds within and between the participating strands, but destroy the binding site for metal cations by changing the orientation of the adjacent carboxyl group. This suggests that no cation-mediated aggregation occurs directly with high acyl gellan, but any salt that may be present within the initial high acyl gellan sample composition is likely to be forced to order and aggregate within the gel matrix. This additional salt ordering at 100% high acyl gellan contributes to the overall high viscosity observed in Fig. 3, whereby a considerably more aggregated, true gel form exists consisting of a larger number of elastically active network chains and junction zones than the remaining mixed gellan solutions. The small plateau in viscosity observed at 100% high acyl gellan at the end of the low temperature transition, also suggests that a threshold concentration for formation of the true high acyl gel has been reached and that prior to this progressive suppression of the electrostatic repulsion between the gellan double helices was taking place.

**Fig. 4:**
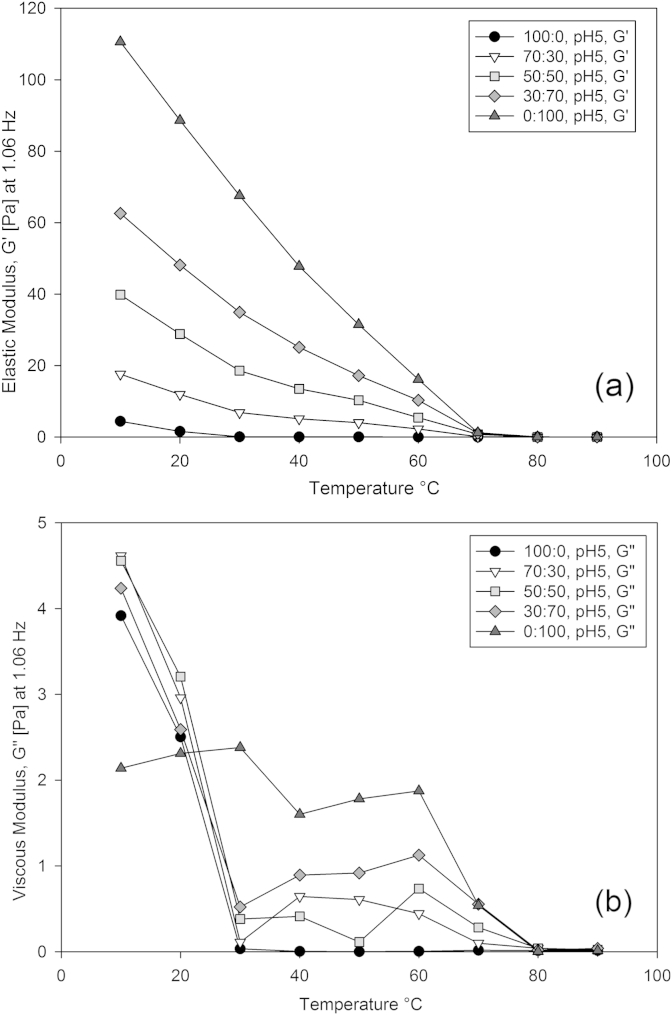
Elastic modulus (a) and viscous modulus (b) measurements at 1.06 Hz versus temperature for 0.5 wt.%, pH5 mixed gellan aqueous solutions as a function of increasing high acyl gellan proportion (0, 30, 50, 70, and 100%) during a temperature ramp (90–10 °C), whilst performing frequency sweeps (0.1–10 Hz) at 10 °C intervals.

Stress controlled oscillation measurements were performed at 0.1 Pa from 90 to 10 °C on the same range of samples as the viscometric tests, by performing frequency tables from 0.1 to 10 Hz at 10 °C intervals. Fig. 4a and b show the resulting elastic and viscous modulus versus temperature measurements for 0.5 wt.%, pH5 mixed gellan aqueous solutions.

Fig. 4a shows that increasing the proportion of HA variant causes an increase in elastic modulus. This increase begins at 70 °C and continues with further cooling, a consequence of the HA coil helix transition. This behaviour is not apparent at 0% HA, and there is no apparent change in elastic modulus due to the LA variant, which would show as a change in value at around 25 °C.

The viscous modulus shown in Fig. 4b shows a sharp increase beginning at 80 °C, and increasing in magnitude with increasing HA proportion, again corresponding to the HA coil-helix transition. In all the gels containing the LA variant there is a further, much larger increase in viscous modulus at 30 °C corresponding to the LA coil-helix transition. This highlights the different characters of the two variants, the LA having an inelastic brittle gel, and the HA a more elastic and less stiff behaviour.

**Fig. 5:**
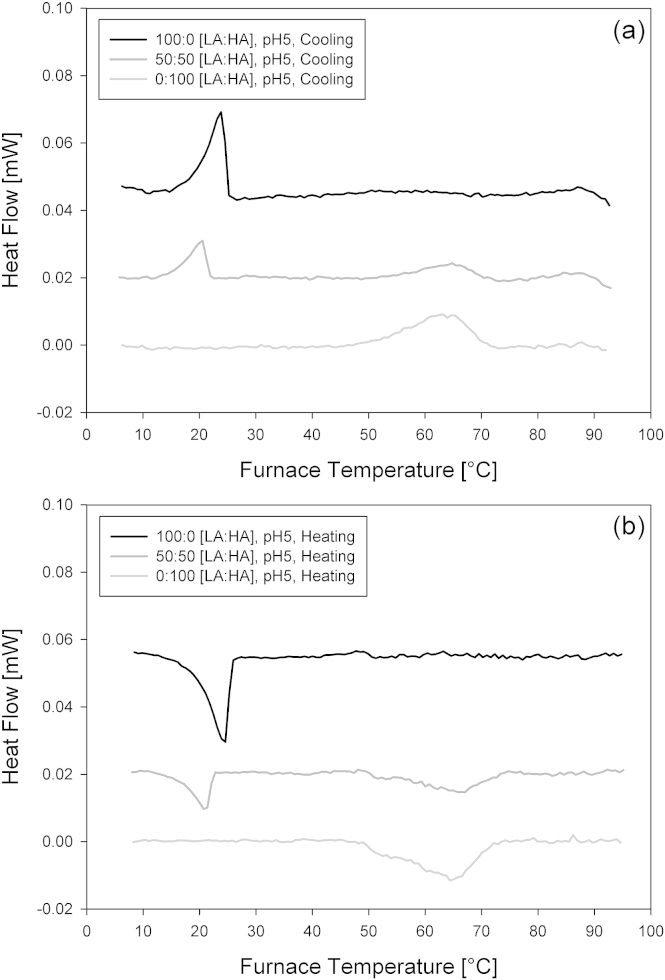
a and b. μ-DSC exothermic (a) and endothermic (b) peaks (baseline subtracted) for 0.5 wt.% mixed gellan gum solutions with 0, 50, and 100% high acyl gellan, at their natural pH. A cooling and heating rate of 0.2 °C/min was implemented. Note that heat flow values have been added and subtracted to separate the curves from each other for clarity purposes.

Fig. 5a and b show the cooling and heating, exothermic and endothermic μ-DSC curves for the 0.5 wt.% mixed gellan gum solutions with 0, 50, and 100% high acyl gellan, at their natural pH values using a scanning rate of 0.2 °C/min.

The low acyl gellan cooling curve (Fig. 5a) showed a single exothermic peak at 23.7 °C, and the heating curve showed a single endothermic peak at 24.3 °C. These two peaks both coincide with the low acyl gellan coil-helical temperature transition observed with the viscosity measurements at the same total gellan concentration in Fig. 3. At 100% high acyl gellan, the cooling curve showed a single exothermic peak at 62.6 °C, and the heating curve showed a single endothermic peak at 64.3 °C. No thermal peaks were measured at around 25 °C for high acyl gellan, which could correspond to the low temperature viscosity transition observed in Fig. 3. The exothermic and endothermic peaks observed in Fig. 5a and b are attributed to the coil-helix transitions of the gellan gum molecules and the subsequent aggregation of these helices. This is generally supportive of what has previously been reported for gellan gum gels in the presence of small amounts of monovalent cations (Miyoshi, Takaya, & Nishinari, 1996).

Kasapis et al. (1999) and Morris, Gothard, Hember, Manning, and Robinson (1996) also reported two separate coil-helix transitions observed by DSC of blends of high acyl and deacylated gellan gum that were coincidental with the peak positions for the individual constituents. Morris, Nishinari, and Rinaudo (2012) later stated that this was firm evidence that high acyl and deacylated gellan do not form double helices incorporating strands of both types.

### 3.2 Post production exposure of gels to an acidic environment

Mixed gellan gels were soaked in 0.5% (0.137 mol/dm^3^) HCl acid for varying time periods. This simulated stomach conditions during digestion and allowed investigation of the effects of prolonged acid exposure on the mechanical properties of the mixed gellan gels after formation at their natural pH.

**Fig. 6:**
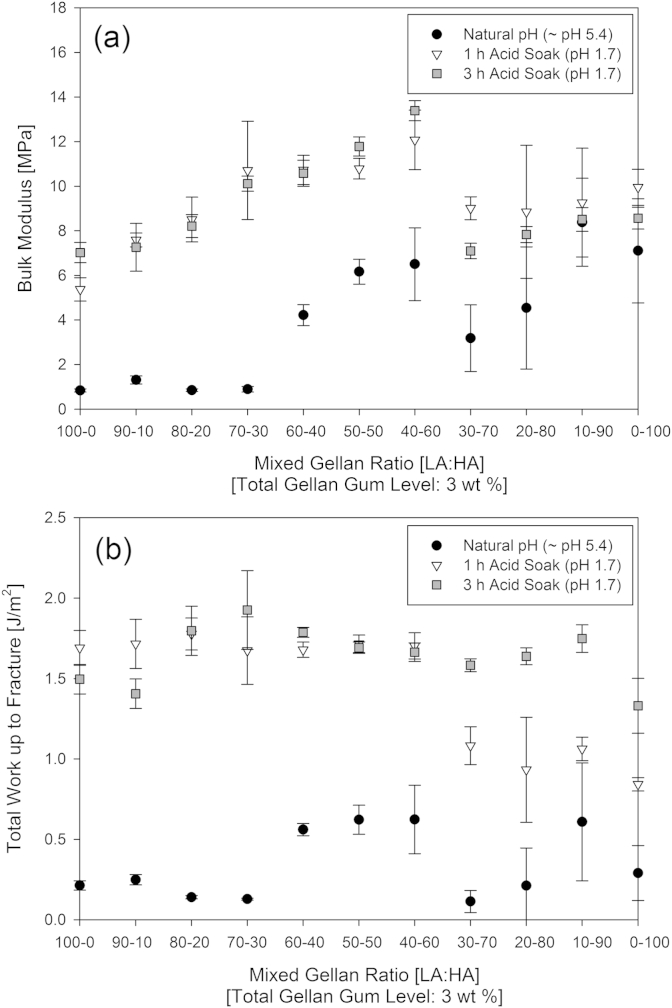
The bulk modulus and work up to fracture data for mixed gellan gels after production, and after exposure to acid.

Fig. 6 shows that exposing the gels to an acidic environment altered their mechanical properties. Irrespective of the low to high acyl gellan weight fractions in the mixtures, the total work to fracture the gels increased after one hour of exposure to acid. This suggests that the cross-links between the hydrocolloid chains are reinforced by acid exposure. Gels containing higher proportions of high acyl gellan take longer in the acid soak to achieve maximum work to fracture, although the bulk modulus decreases between one and three hours exposure to acid. The step change in the bulk modulus between 60 and 70% high acyl gellan, suggests a change in which variant is forming the continuous matrix. The smooth linear increase of moduli between 0 and 60% high acyl gellan after one hour of acid exposure indicates a semi-interpenetrating network structure. The low acyl gellan forms a continuous gel matrix in which, small amounts of the high acyl gellan are dispersed, as opposed to the phase-separated structure seen at natural pH. This once again follows from the observations of Morris et al. (2012) that when there is no indication of discontinuity in properties (as would be expected from phase separation), an interpenetrating network structure is dominant. Above 60% high acyl gellan, the network formation remains semi-interpenetrating, but the opposite structure is suggested, with discreet low acyl gellan polymers being dispersed in a continuous high acyl gellan matrix as suggested by Mao, Tang, and Swanson (2000). With each of these proposed network structures, only the continuous phase gellan polymer chain is cross-linked, with the other remaining linear.

It is widely reported that the low acyl gellan is acid sensitive, whilst the high acyl gellan form is not (Norton et al., 2011; Yamamoto & Cunha, 2007). Thus, it may be questioned why the postproduction acid exposure of the mixed gellan is causing the high proportion high acyl gel strength to increase at all. One possible explanation is that the _L_-glycerol groups in high acyl gellan increase the stability of the double helix by forming additional hydrogen bonds within and between the participating strands, but remove the binding site for metal cations by changing the orientation of the adjacent carboxyl group (Morris et al., 2012). The consequent loss of cation-mediated aggregation reduces gel strength, but makes the high acyl gellan more susceptible to the positively charged protons from the acid, encouraging the formation of additional hydrogen bonds resulting in an overall gel strength increase (Horinaka, Kani, Hori, & Maeda, 2004; Miyoshi et al., 1996; Miyoshi, Takaya, & Nishinari, 1998; Nickerson, Paulson, & Speers, 2003; Tang, Tung, & Zeng, 1997; Yamamoto & Cunha, 2007).

Increasing the acid exposure time from one hour to three hours does not seem to further affect the structure or mechanical properties of the gels that contain a large proportion of low acyl gellan. The gels with a higher proportion of high acyl gellan however, are beginning to show signs of weakening structure after exposure for three hours, with the bulk modulus values beginning to fall. This effect is possibly caused by the acyl groups becoming charged at low pH, and repelling each other. This in turn causes a conformational change in the glycosidic bond between sugars, exposing the oxygen to acid attack and subsequent hydrolysis.

### 3.3 Acid exposure during gelation

**Fig. 7:**
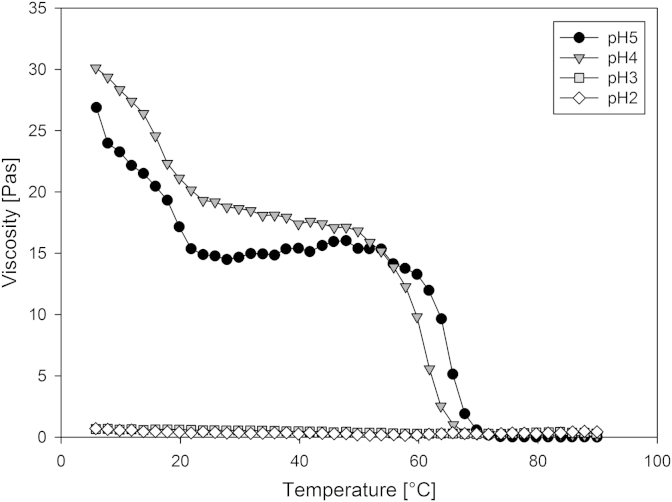
Viscosity measurements of 0.5 wt.%, 70% high acyl mixed gellan aqueous solutions at varying pH's during a temperature ramp (90–5 °C) at 2 °C/min, 0.5 s^−1^ constant shear rate.

Adjusting the acidity of the mixed gellan solutions via the direct addition (dropwise) of 0.5 wt.% (0.137 mol/dm^3^) HCl at 80 °C during their production, altered the measured viscosity during network formation. Fig. 7 shows the viscosity measurements of 0.5 wt.%, 70% high acyl mixed gellan aqueous solutions at varying pH's, over a range of temperatures under a constant shear rate of 0.5 s^−1^.

At pH 4 and 5 we observed the two high and low temperature coil-helix transitions of the high acyl gellan gum and low acyl gellan gums respectively, as also presented in Fig. 3. However, at pH3 – 2, no structuring was evident across the temperature range. This same trend was also observed with the 0%, 30%, 50% and 100% proportion high acyl mixed gellan solutions.

The gelation of gellan can be induced by the reduction in pH, with Grasdalen and Smidsrød (1987) describing HCl as ‘the most potent gel-former’. However, the variation in gel strength with increasing concentration of acid is not monotonic. Initial acidification from neutral pH to pH 3.5 causes a large increase in work to fracture (Picone & Cunha, 2011). On further decrease in pH below the pK_a_ of the glucuronate residues in gellan at approximately pH 3.4 (Haug, 1964), work to fracture decreases (Norton et al., 2011). At pH2 the gels are extremely weak and turbid, and show phase separation of polymer and solvent (Moritaka, Nishinari, Taki, & Fukaba, 1995).

A possible explanation for the flat viscosity observed at acidic pH's in Fig. 7 is that the shear rate used to measure the viscosity of the mixed gel solutions is subsequently causing the gel to break during its formation or is preventing it from forming. Alternatively sheared gels could be being produced at these low pH values. If sheared gels were produced at these low pH values, we would still observe some increase in viscosity. The fact that we do not suggests that sheared gels are unlikely to be responsible for the constant viscosity trends observed.

**Fig. 8:**
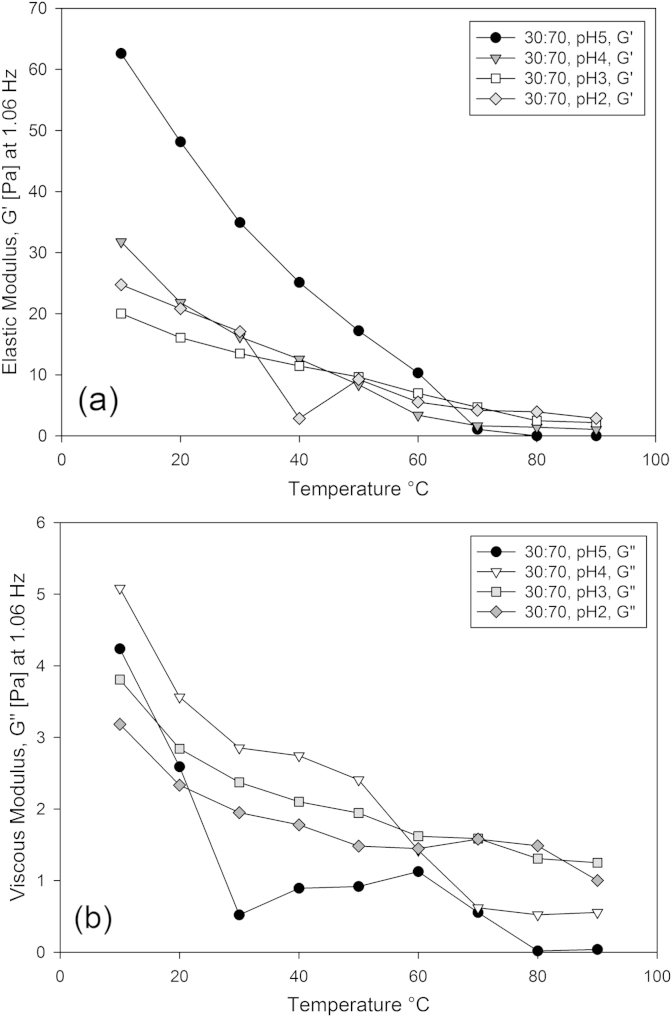
Elastic modulus (a) and viscous (b) measurements at 1.06 Hz versus temperature for 0.5 wt.%, 70% high acyl mixed gellan aqueous solutions as a function of pH (natural – 2) during a temperature ramp (90–10 °C), whilst performing a frequency sweep (0.1–10 Hz) at 10 °C intervals.

Adjusting the acidity of the mixed gellan solutions via the direct addition (dropwise) of 0.5 wt.% (0.137 mol/dm^3^) HCl at 80 °C during their production, also had an impact on the elastic and viscous moduli during network formation. Fig. 8a and b show the elastic and viscous moduli versus temperature measurements for 0.5 wt.%, 70% proportion high acyl mixed gellan aqueous solutions as a function of pH over a range of temperatures at constant stress. Fig. 8 shows that with lower pHs, the elastic and viscous moduli increased on cooling, despite no observed changes in the corresponding viscosity measurements. As was observed with the un-acidified gels, only a single increase in the elastic modulus was observed at 70 °C at pH5, corresponding to the high acyl gellan coil-helical transition, instead of the two transitions present in the viscosity data for the respective pH value. This is attributed to the elastic and non-elastic natures of the high acyl and low acyl gellan gels respectively.

Fig. 8b shows the two distinct regions of increase in the viscous modulus coincidental with the high acyl and low acyl gellan coil-helical transitions for pH 4 and 5 at approximately 75 °C and 30 °C respectively. The low acyl gellan low temperature transition becomes less pronounced as the pH is lowered. At low pH values, no significant rises in the viscous modulus were observed at a specific temperature. A steady increase in the viscous modulus with decreasing temperature was observed in a similar fashion to the elastic modulus.

Direct addition of HCl acid to the natural pH mixed gellan aqueous solutions in this way is a fast method of acidification that is novel for biopolymers of this type, where slower and alternative methods using different acids have been investigated. Alginate acidification has been undertaken by slow exposure of alginate to a D-glucono-delta-lactone produced acid environment (Draget et al., 2006), where acid gels are produced progressively during a two-hour period. The rate of aggregation using this direct HCl addition method is expected to be much higher than the rate achieved by thermally induced gelation. This suggests that the extent of cross-linking between the polymer chains in the case of direct addition of HCl becomes lower than when cross-linking occurs in the case of thermally set gels. This results in altered elasticity and strength of the overall mixed gellan acid structure.

For each of the pHs tested, it was the 50% high acyl mixed gellan solutions that produced the gels with the highest bulk moduli and work to fracture. Similar behaviour has been reported by Mao et al. (2000), who suggest that a strong synergistic interaction may exist between low acyl and high acyl gellan at 50:50 ratio, where each of the hydrocolloids form continuous networks separately, resulting in strong full-interpenetrating network structures, where both polymers are self cross-linked.

### 3.4 Effect of biopolymer concentration

**Fig. 9:**
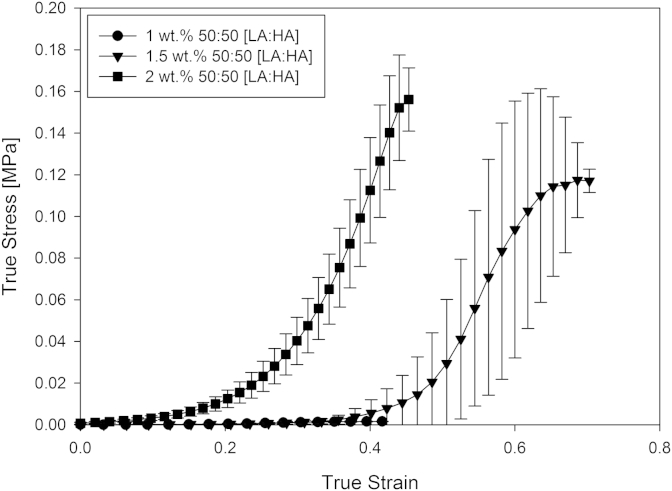
True stress/true strain curves for the 50% high acyl mixed gellan gels at pH4, as a function of increasing gellan concentration (1–2 wt.%).

**Fig. 10:**
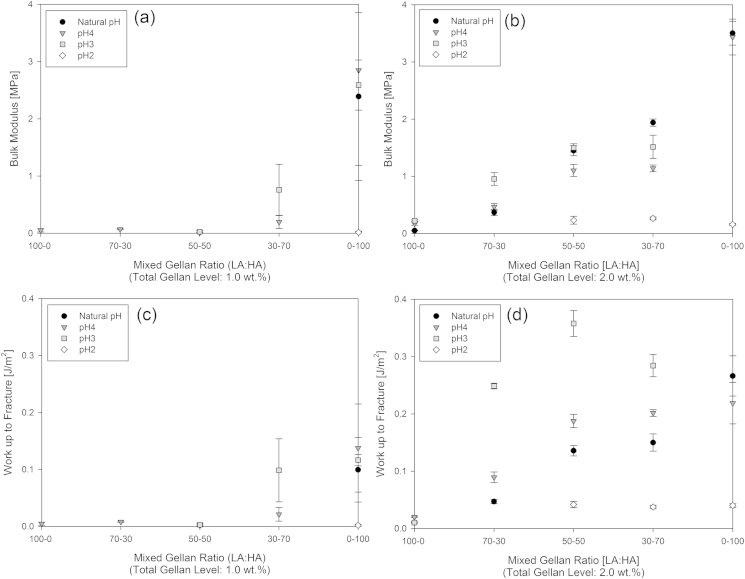
Bulk modulus (a–b) and work up to fracture (c–d) for the mixed gellan gels, as a function of pH at gellan concentrations 1 wt.% and 2 wt.% respectively, each stored at 5 °C. Note, where data plots are missing, the gels formed were not strong enough to be tested.

Addition of acid to the mixed gellan solutions during their production has a significant influence on their resulting rheological properties. These physical properties of the formed mixed gellan acid gels were also found to depend on the total polymer concentration in the systems. Fig. 9 shows the true stress/strain curves for the 50% high acyl mixed gellan gels at pH4, as a function of increasing gellan concentration (1–2 wt.%) and the bulk modulus and work of fracture values calculated from this data are plotted in Fig. 10.

The bulk moduli and work to fracture both increase as the concentration of the gellan in the mixed acid gels is increased. Each of the mixed gel samples displayed purely brittle fracture behaviour, with a rapid decrease in the applied stress once the gels fail at strains typically between 0.4 and 0.45, where a clear fracture point is observed. This behaviour is observed for the gellan concentrations 1 and 2 wt.% in Fig. 9, however a shifted failure strain is visible for the 1.5 wt.% gellan concentration. Shift in strain values are generally indicative of an increase in gel brittleness (strain at break).

Both Figs. 9 and 10 display concentration dependency, that with increasing biopolymer concentration, firmer gels are produced as a result of the increased extent of interactions occurring between the hydrocolloid chains.

Increasing the total biopolymer concentration normally increases gel firmness, increasing the absolute amount of high acyl gellan in the mixed gels also has a significant impact on the corresponding gel firmness. Fig. 10 shows the data for the 1 wt.% and 2 wt.% mixed gellan acid gels, indicating that the proportion of each hydrocolloid is more influential over the mechanical properties than total biopolymer concentration. Irrespective of total biopolymer concentration the gels with a higher high acyl proportion retain their mechanical properties better at low pH. The gels show an increase in bulk modulus as more of the high acyl gellan is added and the mixed gellan acid gels become more turbid as the proportion of high acyl gellan is increased, demonstrating the presence of highly aggregated structures.

Fig. 10 shows that adjusting the acidity of the 1 wt.% and 2 wt.% mixed acid gels during their production has an influence on the resulting gel properties. The natural pH of the gellan solutions was measured at 5.4 and this was not dependent upon the gellan concentrations used. Mixed acid gels with pH's 3–4 were more resistant to fracture than those at pH2. Although some overlap was observed between the natural pH, pH4, and pH3 calculations for the 1 wt.% mixed gellan gels, presumably due to the greater difficulty in molecular ordering within their weak gel environments. This demonstrates that the gels become more resistant to deformation as a result of greater numbers of cross-links between the hydrocolloid chains promoted at the lower pH conditions.

Control of the physical properties of mixed gellan acid gels can be implemented by changing their biopolymer concentration, and proportion of HA and LA types, but it is this HA:LA ratio that is the most influential.

## 4 Conclusions

Mixes of high and low acyl gellan variants produce gels with properties intermediate to either variant alone.

The mixtures display a discontinuous change in gel properties such as bulk modulus as the proportions of low acyl and high acyl gellan are varied showing a phase-separated gel structure. This is further shown using DSC analysis, which shows separate conformational transitions for the two variants at natural pH.

Acid can be used to affect gelation and gel properties of the separate and mixed low and high acyl gellan gels. The resultant gel structure becomes semi-interpenetrating rather than phase separated, as shown by the continuous change in properties with changes in proportion of the two variants.

High acyl gellan is less affected by acid exposure than the low acyl variant, with less change in bulk modulus with acid at higher high acyl gellan proportions. The low acyl variant produces more brittle (having higher work to fracture) gels with lower bulk modulus. The mixed gels combine these properties, producing gels that have higher bulk modulus under acidic conditions without becoming brittle.

Direct acidification during gelation produces a variation in bulk modulus and work to fracture of the gels, with phase separation of the gel and the solvent being evident for all the high acyl to low acyl proportions at pHs less than 3. The highest bulk modulus achieved was at pH3 with 50% of each of the gellan variants. Increasing the proportion of the low acyl variant at low pH during gelation causes a large reduction in bulk modulus and work to fracture; the high acyl variant is much more resistant to the effects of acid during gelation.

The total biopolymer concentration of the gel has an effect on the gel strength, but the relative proportion of low to high acyl gellan variants also has a significant effect.

Mixed gellan gels could be used as an ingredient in foods to structure the stomach contents via interaction with the acidic environment of the stomach. Prolonged exposure to acid starts to reduce the bulk modulus of mixed gellan gels with a large high acyl gellan proportion (>60%).

### 4.1 Further study

The storage of these gels may affect their properties (although no significant changes were recorded during this study for up to a week of storage at room temperature). Before they can be used as satiety increasing agents in formulation of foods this needs to be further investigated, along with the effects of excess water and how acidification changes their water holding capacity.

The results shown here would be further corroborated by microscopy showing the distribution of the two gellan types within the mixed system. This presents difficulties however, because the two biopolymers have such similar structures they cannot be differentiated using conventional staining techniques, and would need to be covalently labelled.
